# Implications of the 2025 AHA/ACC high blood pressure guidelines on the initiation and intensification of blood pressure-lowering medications among US adults

**DOI:** 10.1016/j.ajpc.2025.101400

**Published:** 2026-01-18

**Authors:** Ahmed Sayed, Eric D. Peterson, Ann Marie Navar

**Affiliations:** a Rochester General Hospital, Rochester, NY, USA; b Department of Medicine, Division of Cardiology, University of Texas Southwestern Medical Center, Dallas, TX, USA

**Keywords:** Hypertension, Blood pressure, Guidelines

## Abstract

**Background:**

Recent updates to the American College of Cardiology (ACC) and American Heart Association (AHA) guidelines for high blood pressure (BP) changed the risk calculator recommended, lowered the preferred treatment target, and expanded treatment recommendations for lower risk adults. We sought to quantify the clinical implications of these change among US adults.

**Methods:**

Using data from the 2015–2020 National Health and Nutrition Examination Survey (NHANES), we estimated the proportion and number of US adults aged 20 years or older who were eligible for initiation or intensification of pharmacological anti-hypertensive medications under the 2017 vs the 2025 guidelines.

**Results:**

Among US adults ≥20 years not being currently treated for hypertension (*N* = 180.0 million), using the 2017 Guideline, 18.7 % (33.6 million) were eligible for initiation of pharmacological anti-hypertensive therapy. In contrast, the 2025 Guideline would treat 18.4 % (33.2 million) with upfront medication while an additional 10.8 % (19.4 million) would be considered for medications if lifestyle modification proves insufficient. Increases in treatment eligibility were most pronounced among younger adults age 30–60 and those with obesity. Among adults currently being treated for hypertension (*N* = 58.0 million), most (59.8 %; 34.6 million) did not meet the recommended goal of a BP <130/80. An additional 17.6 % (5.6 million) are newly eligible for treatment intensification if pursuing the preferred BP target of <120/80.

**Conclusion:**

The new 2025 AHA/ACC Hypertension Guidelines potentially expands the number of adults eligible for initiation of antihypertensives, particularly in persons who are young and/or obese, and markedly expands number eligible for intensification.

## Graphical abstract

**Figure:**
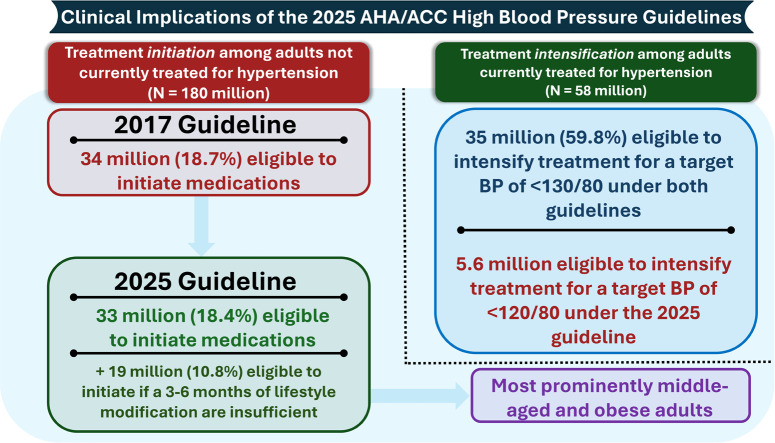

## 1 Introduction

Elevated blood pressure remains a leading cause of morbidity and mortality in the US and worldwide [1]. The newly-introduced 2025 American College of Cardiology/American Heart Association (AHA/ACC) guidelines on the management of high blood pressure in adults contain a number of potentially important changes compared to its 2017 predecessor [2,3]. The most prominent modifications include the use of the Predicting Risk of Cardiovascular Disease Events (PREVENT) equations rather than the Pooled Cohort Equations (PCE) for risk assessment, the use of total cardiovascular disease (CVD) rather than atherosclerotic cardiovascular disease (ASCVD)-based risk estimates, a new recommendation for pharmacological treatment of lower-risk adults with stage 1 hypertension if lifestyle changes prove insufficient, and a preference for a blood pressure (BP) target of <120/80 rather than <130/80 where clinically feasible.

These changes may materially expand the number of US adults eligible for initiation of pharmacological therapy and also the number of currently-treated US adults eligible for treatment intensification to achieve the new preferred BP targets. Accordingly, we sought to describe the implications of the new recommendations on the management of hypertension among US adults and identify the demographic subgroups where these recommendations are most impactful.

## 2 Methods

### 2.1 Data source and study population

Publicly available data from 2 consecutive cycles (2015–2020) of The National Health and Nutrition Examination Survey (NHANES), a nationally representative random sample of ambulatory US adults, were used for this analysis [4]. Participants were interviewed regarding demographics, medical conditions, and underwent medical examinations and laboratory assessments. All adults aged ≥20 years were eligible for the present analysis. Because BP was taken as the average of 3 measurements, participants were excluded if they had <3 valid systolic and diastolic BP (SBP and DBP, respectively) measurements (*N* = 1333 excluded) or insufficient data to determine guideline-based treatment recommendations (*N* = 1144 excluded). NHANES protocols were approved by the National Center for Health Statistics Ethics Review Board and all NHANES participants provided informed consent. The current analysis used de-identified publicly-available data and was thus exempt from Institutional Review Board review.

### 2.2 Outcomes

The two outcomes of interest were the proportion of adults eligible for initiation of BP-lowering medications (among adults reporting no current use of pharmacotherapy for hypertension) and the proportion of adults eligible for intensification of BP-lowering medications (among adults reporting current use of pharmacotherapy for hypertension).

*A. Eligibility for initiation*

Criteria for treatment initiation in the present analysis are shown in **Supplementary Table 1**. Per the 2025 guidelines, eligibility for initiation (class I recommendation) was determined by a BP ≥140/90 mm Hg or a BP ≥130/80 in the setting of elevated 10-year risk of cardiovascular disease (≥7.5 % by base PREVENT), diabetes (defined by an HbA1c ≥6.5 %, fasting glucose ≥126mg/dL, or a self-reported history of diabetes), chronic kidney disease (CKD; defined by an eGFR ≤60 mL/min/1.73 m² or a urine albumin-creatinine ratio ≥30mg/g), or established cardiovascular disease (defined as a self-reported history of angina, coronary heart disease, myocardial infarction, stroke, or heart failure) [2]. An additional analysis was performed wherein an additional criteria (≥130/80 mm Hg in the absence of the aforementioned risk factors) was included because the guidelines recommend pharmacological treatment in this subgroup of patients if 3–6 months of lifestyle modification does not reduce BP to <130/80 mm Hg [2]. Proportions using both definitions are presented. For the primary analysis, the base PREVENT equation was used. A sensitivity analysis using the expanded PREVENT equation to additionally include HbA1c and urine albumin-creatinine ratio was conducted.

Per the 2017 guidelines, eligibility was determined by a BP ≥140/90 mm Hg or a BP ≥130/80 mm Hg in the setting of a PCE 10-year risk of ASCVD ≥10 %, diabetes, CKD, established CVD, or an SBP ≥130 mm Hg for older adults (≥65 years) [3]. Risk estimates by PREVENT and PCE were only calculated for adults aged 30 to 79 years and 40 to 79 years respectively, as these were the age-groups in which these equations were intended for use. For persons outside these age ranges, treatment recommendations were based on BP only.

In addition to determining the proportion eligible by either guideline, the proportions with concordant or discordant recommendations are also presented. The association of age, sex, race, and body mass index (BMI) with discordance was assessed using logistic regression models. For the two continuous variables assessed (age and BMI), the proportion eligible for treatment using either guideline was estimated across their spectra individually and in combination. The association of CVD, DM, and CKD with discordance was not assessed because both guidelines share the same 130/80 threshold for initiation in these subgroups.

*B. Eligibility for intensification*

Eligibility for treatment intensification was divided according to 3 categories: BP ≥130/80 mm Hg (eligible for intensification by both guidelines), SBP of 120–129 mm Hg and DBP <80 mm Hg (eligible for intensification by 2025 guidelines to achieve a preferred target of <120/80 mm Hg), or a BP <120/80 mm Hg (well-controlled). Subgroup analyses were performed by age (<65 and ≥65 years), sex (males and females), race/ethnicity (Non-Hispanic [NH] Black, White, Asian, or Hispanic), and comorbidities (obesity, diabetes, CVD, and CKD). Because BP goal recommendations are stronger for higher-risk vs lower-risk adults (1A vs 2b respectively), [2] an additional subgroup analysis stratified by 10-year ASCVD risk by PREVENT (≥7.5 % vs <7.5 %) was also performed. Patients with CVD or CKD were analyzed separately as the guidelines do not explicitly endorse a target of <120/80 mmHg for these patients.

### 2.3 Statistical analysis

Eligibility for treatment initiation or intensification is presented as simple proportions ( %) and the equivalent number of US adults (millions). To assess variables associated with discordant recommendations for treatment initiation, logistic regression models were used with discordance as the outcome variable. For age and BMI specifically (continuous variables which were associated with discordance), the proportion eligible for treatment by either guideline across the full spectra of each variable was estimated using restricted cubic splines with 5 knots. The joint association of age and BMI with changes in treatment eligibility was assessed using a tensor spline of the two restricted cubic splines.

NHANES examination weights were used to account for the survey design. Combining survey weights of the 2017–2020 and 2015–2016 survey cycles was done in accordance with published NHANES analytic guidelines [5]. To adjust for item nonresponse (that some survey respondents did not answer questions or have measurements necessary to determine their eligibility), the survey weights of adults with non-missing data were adjusted using their response propensities [6] Response propensities for each participant were estimated using a survey-weighted logistic regression model containing age, sex, and race as predictor variables. Survey weights were then adjusted by the inverse of the predicted response probabilities. This allowed us to make inference regarding the target population (US adults aged 20 years or older) despite data missingness while also serving to mitigate biases that may arise due selective non-response. Analyses were performed on R, version 4.4.2 (R Foundation for Statistical Computing, Vienna, Austria) using the “survey” package [7,8]. Statistical significance was denoted by a two-tailed *P* < 0.05 or a 95 % confidence interval excluding the null.

## 3 Results

### 3.1 Baseline characteristics

A total of 11,711 participants aged 20 years or older were included in this analysis. Of these, 6310 reported a history of hypertension, of whom 3336 reported the use of pharmacological BP-lowering medications. The median age (25th – 75th percentile) was 47 years (33 to 62 years), 51.9 % were females, 41.3 % had obesity, 15.2 % had diabetes, 14.3 % had CKD, and 9.3 % had CVD.

## 4 Eligibility for initiation of pharmacological treatment

A. Number of eligible adults under both guidelines

**Fig. 1:**
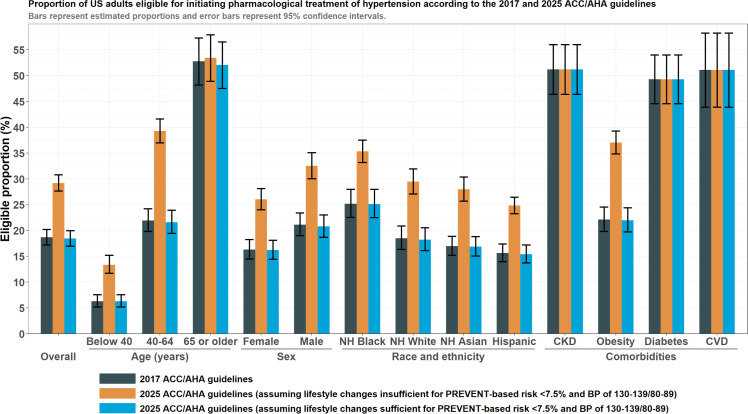
Proportion of US adults eligible for initiating or intensifying pharmacological treatment of hypertension according to the 2017 and 2025 AHA/ACC guidelines. Bars represent estimated proportions and error bars represent 95 % confidence intervals. For the 2025 guidelines, two sets of estimates depending on whether adults with a low PREVENT-based 10-year risk (i.e., <7.5 %) and stage 1 hypertension are able to sufficiently reduce blood pressure to below 130/80 mm Hg.

A total of 180.0 million US adults aged ≥20 years did not report use of pharmacological BP-lowering. Of these, 18.7 % [95 %CI: 17.2 to 20.2 %], representing 33.6 million adults, were eligible for initiation of pharmacological BP-lowering according to the 2017 guidelines (Table 1 & Fig. 1). According to the 2025 guidelines, 18.4 % [95 %CI: 17.0 to 20.0 %], representing 33.2 million adults, were eligible for immediate initiation of pharmacological BP-lowering, such that 0.4 million fewer adults would be eligible for immediate initiation of BP-lowering medications. If treatment is initiated for lower-risk individuals with a BP of 130–139/80–89 mm Hg wherein a 3–6 month trial of lifestyle modification is insufficient, the proportion eligible by the 2025 guidelines would increase to 29.2 % [95 %CI: 27.6 to 30.8 %], representing 52.6 million adults, such that 19.0 more million adults would become eligible for initiation of BP-lowering medications. Sensitivity analyses using the expanded version of PREVENT (to include HbA1c, the urine albumin-creatinine ratio, and both) yielded similar results and are shown in **Supplementary Figure 1–3**.

B. Frequency of concordant and discordant recommendations

**Table 1:** US adults not currently taking medications to lower blood pressure who are eligible for pharmacological treatment, according to the 2017 and 2025 AHA/ACC guidelines.

		Eligible for treatment by the 2017 ACC/AHA guidelines		Eligible for immediate treatment by the 2025 ACC/AHA guidelines					Eligible for treatment by the 2025 ACC/AHA guidelines if lifestyle modification proves insufficient to reduce BP <130/80			
Variable	Strata	Proportion [95 % CI], %	Number [95 % CI], millions		Proportion [95 % CI], %	Number [95 % CI], millions	Difference vs 2017 guidelines, % [95 % CI]	Difference vs 2017 guidelines, millions [95 % CI]	Proportion [95 % CI], %	Number [95 % CI], millions	Difference vs 2017 guidelines, % [95 % CI]	Difference vs 2017 guidelines, millions [95 % CI]
Overall		18.7 [17.2 to 20.2]	33.6 [31.1 to 36.1]		18.4 [17.0 to 20.0]	33.2 [30.6 to 35.7]	−0.2 [−0.4 to −0.1]	−0.4 [−0.7 to −0.1]	29.2 [27.6 to 30.8]	52.6 [49.2 to 56.0]	10.5 [9.6 to 11.4]	19.0 [17.3 to 20.6]
Age (years)	Below 40	6.3 [5.2 to 7.6]	5.2 [4.3 to 6.1]		6.3 [5.2 to 7.6]	5.2 [4.3 to 6.1]	0.0 [0.0 to 0.0]	0.0 [0.0 to 0.0]	13.4 [11.7 to 15.2]	11.0 [9.6 to 12.4]	7.1 [6.1 to 8.1]	5.8 [5.0 to 6.7]
	40–64	21.9 [19.8 to 24.2]	16.4 [14.7 to 18.2]		21.6 [19.5 to 23.9]	16.2 [14.4 to 18.0]	−0.3 [−0.6 to −0.1]	−0.3 [−0.5 to 0.0]	39.3 [37.0 to 41.6]	29.4 [26.9 to 31.9]	17.3 [15.5 to 19.2]	13.0 [11.6 to 14.3]
	65 or older	52.8 [48.2 to 57.3]	12.0 [10.3 to 13.6]		52.0 [47.5 to 56.5]	11.8 [10.2 to 13.4]	−0.7 [−1.7 to 0.2]	−0.2 [−0.4 to 0.0]	53.4 [48.9 to 57.9]	12.1 [10.5 to 13.8]	0.7 [0.0 to 1.3]	0.2 [0.0 to 0.3]
Sex	Female	16.3 [14.5 to 18.2]	15.0 [13.0 to 16.9]		16.2 [14.4 to 18.1]	14.9 [12.9 to 16.8]	−0.1 [−0.3 to 0.1]	−0.1 [−0.3 to 0.1]	26.0 [24.0 to 28.1]	23.9 [21.5 to 26.4]	9.7 [8.7 to 10.7]	8.9 [8.0 to 9.9]
	Male	21.1 [19.0 to 23.4]	18.6 [16.8 to 20.4]		20.8 [18.7 to 23.0]	18.3 [16.5 to 20.1]	−0.4 [−0.6 to −0.1]	−0.3 [−0.6 to −0.1]	32.5 [30.0 to 35.1]	28.6 [26.0 to 31.3]	11.4 [10.0 to 12.8]	10.0 [8.8 to 11.3]
Race and ethnicity	NH Black	25.2 [22.5 to 28.0]	4.7 [3.6 to 5.8]		25.1 [22.5 to 28.0]	4.7 [3.6 to 5.8]	0.0 [−0.3 to 0.2]	0.0 [−0.1 to 0.0]	35.3 [33.2 to 37.5]	6.6 [5.2 to 8.0]	10.2 [8.5 to 11.8]	1.9 [1.6 to 2.2]
	NH White	18.5 [16.4 to 20.9]	20.7 [17.9 to 23.5]		18.2 [16.1 to 20.5]	20.4 [17.6 to 23.1]	−0.3 [−0.6 to 0.0]	−0.3 [−0.6 to 0.0]	29.5 [27.1 to 31.9]	32.9 [29.1 to 36.8]	10.9 [9.6 to 12.2]	12.2 [10.8 to 13.7]
	NH Asian	17.0 [15.2 to 18.9]	1.9 [1.5 to 2.4]		16.9 [15.1 to 18.8]	1.9 [1.5 to 2.4]	−0.1 [−0.5 to 0.3]	0.0 [−0.1 to 0.0]	28.0 [25.7 to 30.4]	3.2 [2.5 to 3.9]	11.0 [9.0 to 13.0]	1.2 [1.0 to 1.5]
	Hispanic	15.6 [14.0 to 17.4]	4.9 [3.9 to 5.8]		15.4 [13.7 to 17.2]	4.8 [3.9 to 5.8]	−0.2 [−0.4 to −0.1]	−0.1 [−0.1 to 0.0]	24.8 [23.3 to 26.5]	7.8 [6.5 to 9.0]	9.2 [7.8 to 10.6]	2.9 [2.4 to 3.3]
Comorbidities	CKD	51.2 [46.4 to 56.0]	8.5 [7.5 to 9.5]		51.2 [46.4 to 56.0]	8.5 [7.5 to 9.5]	0.0 [0.0 to 0.0]	0.0 [0.0 to 0.0]	51.2 [46.4 to 56.0]	8.5 [7.5 to 9.5]	0.0 [0.0 to 0.0]	0.0 [0.0 to 0.0]
	Obesity	22.1 [19.8 to 24.5]	14.6 [12.9 to 16.2]		22.0 [19.7 to 24.4]	14.5 [12.9 to 16.1]	−0.1 [−0.4 to 0.2]	−0.1 [−0.3 to 0.1]	37.0 [34.9 to 39.3]	24.4 [22.3 to 26.5]	14.9 [13.4 to 16.5]	9.8 [8.8 to 10.9]
	Diabetes	49.3 [44.6 to 54.0]	7.6 [6.7 to 8.5]		49.3 [44.6 to 54.0]	7.6 [6.7 to 8.5]	0.0 [0.0 to 0.0]	0.0 [0.0 to 0.0]	49.3 [44.6 to 54.0]	7.6 [6.7 to 8.5]	0.0 [0.0 to 0.0]	0.0 [0.0 to 0.0]
	CVD	51.1 [43.9 to 58.3]	4.2 [3.5 to 4.9]		51.1 [43.9 to 58.3]	4.2 [3.5 to 4.9]			51.1 [43.9 to 58.3]	4.2 [3.5 to 4.9]		

ACC: American College of Cardiology; AHA: American Heart Association; CVD: Cardiovascular disease; CKD: Chronic kidney disease.

Treatment initiation recommendation by both guidelines were concordant for most adults: 18.3 % [95 %CI: 16.9 to 19.9 %] had concordant class I treatment recommendations by both the 2017 and 2025 guidelines, whereas 70.8 % [95 %CI: 69.2 to 72.4 %] lacked such an indication by both guidelines. 0.1 % [95 %CI: 0.0 to 0.2 %] adults would be rendered newly eligible for immediate treatment by the 2025 guidelines. Potential discordance with possibility of pharmacological treatment in the new guidelines (if a 3–6 month trial of lifestyle modification proves unsuccessful), was present in 10.5 % [95 %CI: 9.6 to 11.4 %]. Potential discordance with possibility of deferring treatment (if aforementioned trial of lifestyle modification is successful) was present in 0.3 % [95 %CI: 0.2 to 0.6 %].

C. Factors associated with discordant recommendations

Characteristics of respondents, according to the concordance or discordance of treatment recommendations, are shown in Table 2. Participants who were newly eligible if lifestyle modification is insufficient were younger, had higher BMI, and had lower 10-year predicted ASCVD risk as predicted by the PCE or or CVD risk as predicted by PREVENT.

**Table 2:** Characteristics of US adults according to the concordance or discordance of 2017 and 2025 AHA/ACC high blood pressure guidelines for the initiation of pharmacological treatment.

	Concordant		Discordant		
Variable	Ineligible by both guidelines	Eligible by both guidelines	Newly eligible if lifestyle modification insufficient	Newly eligible for immediate treatment	Newly ineligible if lifestyle modification sufficient
Respondents in NHANES, N	5614	1908	806	11	26
US adults, N (millions)	127.5	33.0	18.8	0.1	0.6
Age (years)	39.8 [39.0 to 40.7]	57.1 [55.8 to 58.5]	45.3 [44.6 to 46.0]	63.1 [59.7 to 66.5]	59.6 [56.1 to 63.1]
Women ( %)	53.4 [51.9 to 54.8]	44.8 [40.5 to 49.1]	47.0 [42.5 to 51.6]	68.3 [6.0 to 98.6]	N/A
Race/ethnicity ( %)					
Hispanic	17.2 [14.2 to 20.5]	12.8 [10.2 to 15.8]	14.8 [12.1 to 18.0]	1.2 [0.2 to 6.7]	N/A
Non-Hispanic Asian	6.0 [4.7 to 7.7]	5.6 [4.3 to 7.1]	6.3 [4.7 to 8.2]	6.5 [1.5 to 23.9]	N/A
Non-Hispanic Black	9.8 [7.8 to 12.2]	15.5 [12.3 to 19.5]	11.0 [8.6 to 14.0]	8.0 [1.9 to 27.5]	N/A
Non-Hispanic White	63.2 [58.8 to 67.3]	62.1 [56.6 to 67.3]	64.5 [59.5 to 69.3]	76.9 [43.0 to 93.6]	N/A
Other (including multiracial)	3.9 [3.4 to 4.5]	4.0 [3.1 to 5.3]	3.4 [2.3 to 4.9]	7.5 [0.5 to 59.0]	N/A
Systolic blood pressure (mm Hg)	112.8 [112.4 to 113.2]	143.3 [142.4 to 144.2]	127.1 [126.4 to 127.8]	129.3 [127.6 to 131.0]	128.8 [125.2 to 132.3]
Diastolic blood pressure (mm Hg)	68.3 [67.9 to 68.7]	82.6 [81.6 to 83.5]	81.6 [81.1 to 82.2]	78.2 [72.9 to 83.5]	81.3 [77.8 to 84.8]
BMI (kg/m²)	28.2 [27.9 to 28.6]	30.1 [29.7 to 30.4]	31.2 [30.6 to 31.9]	32.2 [27.9 to 36.4]	28.1 [25.7 to 30.4]
HbA1c ( %)	5.4 [5.4 to 5.5]	6.0 [5.9 to 6.1]	5.5 [5.4 to 5.5]	5.6 [5.4 to 5.9]	5.6 [5.4 to 5.7]
Total cholesterol (mg/dL)	185.9 [184.1 to 187.7]	201.3 [198.4 to 204.2]	204.0 [198.7 to 209.2]	189.2 [159.1 to 219.2]	206.8 [183.0 to 230.7]
HDL cholesterol (mg/dL)	55.1 [54.2 to 56.0]	54.7 [53.3 to 56.1]	54.2 [52.7 to 55.8]	50.3 [37.5 to 63.1]	47.1 [39.2 to 54.9]
Non-HDL cholesterol (mg/dL)	130.8 [129.0 to 132.6]	146.6 [143.5 to 149.6]	149.7 [144.6 to 154.8]	138.9 [114.6 to 163.2]	159.8 [135.2 to 184.3]
PCE 10-year ASCVD risk, %	5.5 [5.1 to 5.9]	16.3 [15.0 to 17.7]	3.2 [3.0 to 3.5]	7.9 [6.3 to 9.5]	9.5 [8.4 to 10.7]
PREVENT 10-year CVD risk, %	3.4 [3.2 to 3.6]	9.2 [8.6 to 9.8]	2.4 [2.3 to 2.5]	8.0 [7.8 to 8.1]	6.5 [6.2 to 6.8]
PREVENT 30-year CVD risk, %	14.2 [13.6 to 14.9]	28.8 [28.0 to 29.6]	14.4 [14.0 to 14.9]	30.1 [28.2 to 32.1]	28.0 [26.8 to 29.2]
Cardiovascular risk factors ( %)					
Obesity ( %)	32.6 [30.3 to 35.0]	44.2 [40.7 to 47.6]	51.8 [46.7 to 56.8]	70.8 [11.1 to 97.9]	N/A
Diabetes ( %)	10.7 [9.8 to 11.7]	31.7 [29.6 to 33.9]	N/A*		
CKD ( %)	9.1 [8.1 to 10.2]	32.6 [30.4 to 34.9]			
CVD ( %)	6.2 [5.3 to 7.2]	20.3 [18.2 to 22.7]			

ACC: American College of Cardiology; AHA: American Heart Association; CVD: Cardiovascular disease; CKD: Chronic kidney disease; PCE: Pooled Cohort Equations; PREVENT: Predicting Risk of Cardiovascular Disease Events.

Values are presented as survey-weighted means (for continuous variables) and proportions (for categorical variables) with 95 % confidence intervals.

⁎ These characteristics warrant initiation of pharmacotherapy in both guidelines even if only stage 1 hypertension is present.

**Fig. 2:**
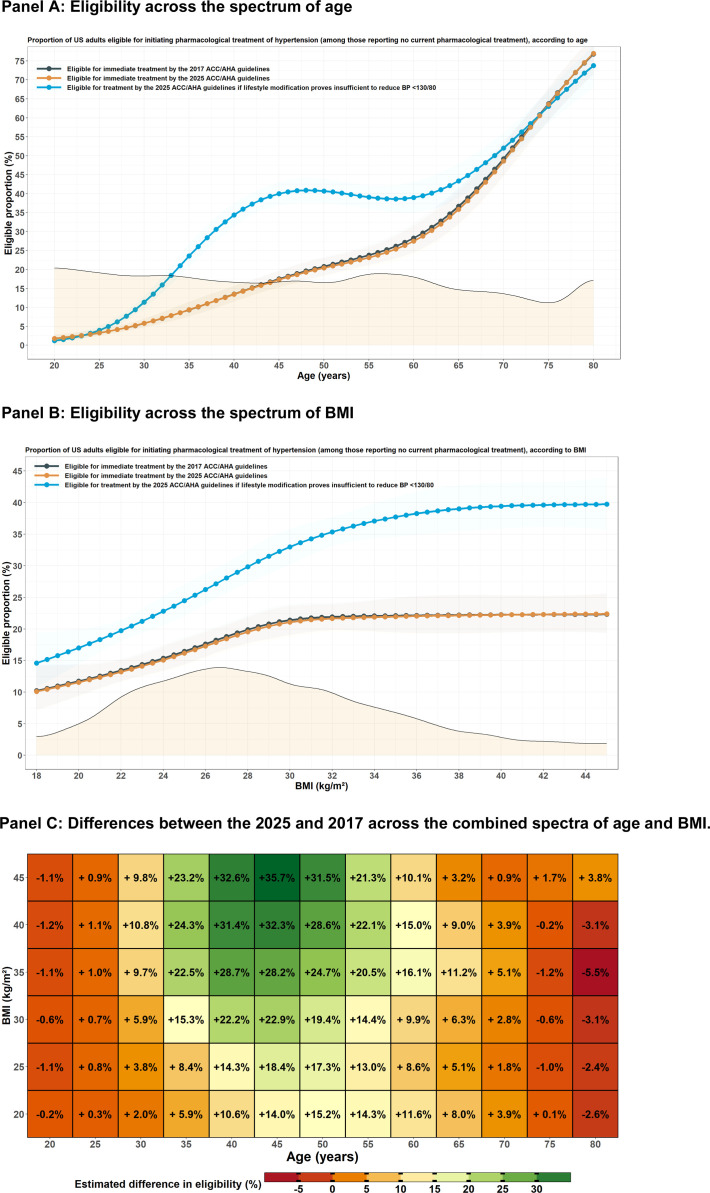
Proportion of concordant & discordant class I recommendations for initiating pharmacological treatment according to the 2025 and 2017 AHA/ACC guidelines. Panels A and B contrast eligibility proportions by each guideline across the spectrum of age and body mass index (BMI), respectively. The shaded area in both panels depicts the weighted distribution of both variables. Panel C shows differences in eligibility between 2025 (assuming insufficiency of lifestyle modification for low-risk stage 1 hypertension) and 2017 across the spectrum of age and BMI. Panel A: Eligibility across the spectrum of age, Panel B: Eligibility across the spectrum of BMI, Panel C: Differences between the 2025 and 2017 across the combined spectra of age and BMI.

Compared with the 2017 guidelines, a higher proportion of middle-aged adults would be eligible for treatment by the 2025 if adults with lower-risk stage 1 hypertension were eligible for eventual treatment due to an unsuccessful 3–6 month trial of lifestyle modification (Fig. 2**A**). For adults aged 40, 50, and 60 years, estimated eligibility with the 2017 vs 2025 guidelines would be 13.5 % vs 34.4 %, 20.8 % vs 40.7 %, and 28.3 % vs 39.0 %, respectively. Conversely, a similar proportion of older adults would be eligible for treatment by both guidelines. For adults aged 70 years, eligibility would be 49.2 % vs 52.0 % respectively.

Additionally, a greater proportion of adults with obesity would be eligible for treatment initiation based on the treatment recommendation for lower-risk stage 1 hypertension (Fig. 2**B**). At a BMI of 30, 35, and 40, the proportion eligible was 21.4 % vs 33.0 %, 22.1 % vs 37.7 %, and 22.3 % vs 39.4 % respectively. Differences by age-BMI were complementary (Fig. 2C).

### 4.1 Eligibility for intensification of pharmacological treatment

**Fig. 3:**
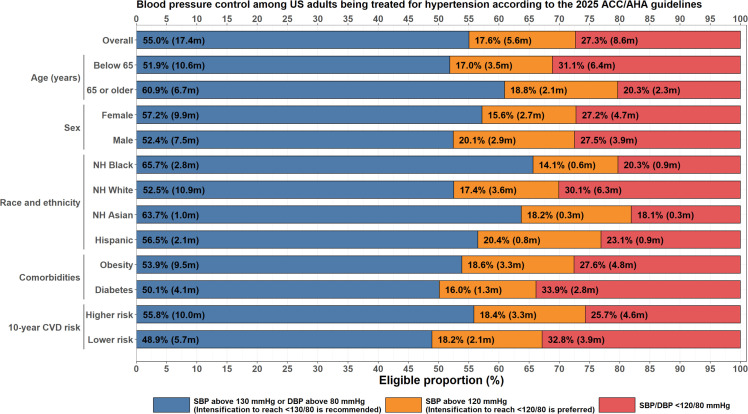
Blood pressure control among US adults being treated for hypertension according to the 2025 AHA/ACC guidelines. Higher 10-year risk is determined by a predicted risk ≥7.5 % by PREVENT. These data exclude patients with established cardiovascular disease (defined as MI, stroke, CAD, or HF) and chronic kidney disease (defined as an ≤60 mL/min/1.73 m² or a urine albumin-creatinine ratio ≥30mg/g), as the 2025 guidelines do not explicitly endorse treatment intensification with a goal of <120/80 mmHg in these subgroups.

A total of 31.6 million US adults aged ≥20 years without CVD or CKD reported use of medications for pharmacological BP-lowering. Of these, 55.0 % [95 %CI: 51.3 to 58.7 %], representing 17.4 million, had a BP of above 130/80 mm Hg; 17.6 % [95 %CI: 14.7 to 20.9 %], representing 5.6 million, had a BP of 120–129/<80 mm Hg; and 27.3 % [95 %CI: 23.1 to 32.0 %], representing 8.6 million, had a BP of <120/80 mm Hg. Older persons (60.9 %), Non-Hispanic Black persons (65.7 %), and Asian persons (63.7 %) had the highest prevalence of BP above 130/80 mmHg (Fig. 3 **and Supplementary Table 2)**. In a sensitivity analysis limited to the 2017–2020 survey cycle (after release of the 2017 guidelines), the proportion of adults with a BP above 130/80 mm Hg was 54.3 % [95 %CI: 50.1 to 58.4 %].

Among adults with CVD receiving antihypertensive medications (*N* = 14.0 million), the proportion with a BP above 130/80 mmHg was 62.5 % [95 %CI: 58.4 to 66.4 %] Among adults with CKD (*N* = 17.0 million), the proportion with a BP above 130/80 mmHg was 66.6 % [95 %CI: 61.3 to 71.6 %]. Overall, among all adults (including those with CVD or CKD) currently being treated for hypertension (*N* = 58.0 million), 59.8 % [95 %CI: 57.7 to 61.9 %] did not meet the recommended BP goal of <130/80.

## 5 Discussion

Depending on the success of lifestyle modification for lower-risk stage 1 hypertension, the effect of the 2025 AHA/ACC guidelines expands eligibility for treatment of hypertension to as many as 19.0 million additional adults. The majority of this expansion occurs in adults with obesity and younger and middle-aged adults. Among those with hypertension on treatment, over half have yet to meet the prior goal of <130/80 mm Hg, and only 1 in 4 are meeting the new preferred target of <120/80 mm Hg.

Most of the expansion in treatment recommendations is driven by the new recommendation to initiate pharmacological treatment in lower-risk (i.e., 10-year risk <7.5 % by PREVENT) adults with stage 1 hypertension in whom a 3–6 month lifestyle intervention is insufficient. Multiple clinical trials have shown that lifestyle modification can result in meaningful reductions in BP, including weight loss, reductions in dietary sodium and alcohol, increases in dietary potassium, and exercise [2,9]. Unfortunately, a large proportion of patients with hypertension report never being counseled on appropriate lifestyle modifications. Further, structural barriers including lack of access to healthy food or safe spaces to exercise, time constraints, and low health literacy pose substantial barriers to effective implementation of lifestyle changes [[10], [11], [12]].

A key finding of this study is that middle-aged adults (age 35–55) experienced the greatest expected increase eligibility for pharmacological treatment assuming a 3-6 month trial of lifestyle changes are insufficient. Under the prior guideline, these adults did not have sufficiently high risk predicted by the PCE to meet criteria to initiate pharmacological treatment. In contrast, the proportion of older adults eligible to initiate pharmacological treatment is nearly identical under both guidelines, likely because an age >65 was considered a risk-enhancing risk factor under older guidelines that merits treatment initiation in stage 1 hypertension regardless of calculated risk. Multiple studies support the long-term, cumulative risk of prolonged exposure to high blood pressure [[13], [14], [15], [16]]. Promoting earlier BP-lowering may reduce this cumulative exposure and thus mitigate the long-term cardiovascular risk posed by elevated BP.

The reason for the increase in eligibility among people with obesity is twofold. First, persons with obesity are more likely to have stage 1 hypertension, which may merit treatment in the new guidelines even with a lower 10-year predicted risk. Second, unlike the PCE, the PREVENT calculator accounts for BMI and produces higher 10-year risk estimates for persons with obesity. An important related development has been the approval and wider use of effective weight-loss medications which also substantially reduce BP in persons with obesity [17,18]. These medications, notwithstanding concerns about wider access and long-term affordability, will complement current use of anti-hypertensive medications and help control elevated BP in a substantial proportion of obese people.

This analysis also highlights the ongoing gaps in hypertension control amongst patients already on therapy. Only around half of patients with hypertension had achieved a BP <130/<80, and around 1 in 4 had achieved the new preferred goal of <120/80. Closing this gap will likely require a multifaceted approach, including public health measures such as wider use of salt-substitutes [19], optimizing use of currently-available medications such as fixed-dose combination pills [20], and the use of novel long-acting treatment options where clinically appropriate [21].

An important novel aspect of the 2025 guidelines is the use of the race-agnostic PREVENT. Previous data has shown that PREVENT yielded lower ASCVD risk estimates than PCE, with the difference being largest among Black persons [22]. These findings lead to concerns that usage of PREVENT would lead to lesser utilization of preventive therapies in Black patients. In the present analysis, the percentage change in eligibility was largely similar between Black and White persons (0 % and −0.3 % respectively). It is important to note that PREVENT-based risk estimates are only relevant for eligibility among adults with stage 1 hypertension, among whom immediate treatment eligibility is determined by 10-year risk estimates. Increases in eligibility by sex were also comparable (−0.1 % and −0.4 % for females and males respectively).

A recent commentary by Zhang and An also sought to analyze the projected impact of the 2025 AHA/ACC guideline, finding 26.8 million additional adults may be eligible to initiate pharmacological measures to lower BP if a trial of lifestyle interventions proves insufficient [23]. Our analysis provides important additional context by identifying that most of this population consists of middle-aged adults, particularly those with obesity. The present analysis also provides data stratified by sex, race/ethnicity, and comorbidity status. Further, we also tackle the burden of above-goal BP among those already taking anti-hypertensive medications, which was beyond the scope of the aforementioned analysis.

### 5.1 Limitations

This analysis has several limitations. First, although BP was taken as the average of 3 measurements, these measurements occurred during a single visit. Second, data from ambulatory and/or home BP measurements, which are recommended by current guidelines, were not available. Third, treatment of hypertension was self-reported, which may introduce misclassification. Fourth, it was difficult to estimate the proportion of adults with lower-risk stage 1 hypertension for whom lifestyle modification would be sufficient, and we therefore could only provide 2 sets of estimates representing the two extreme possibilities. However, the actual proportion likely lies in between these 2 extremes. Further, implementation of the new guideline recommendations in daily practice is likely to be subject to several limitations, including clinical inertia and delays in embedding risk calculators within the electronic medical record. As such, the projections studied here are unlikely to be realized to their full extent. Furthermore, many physicians and patients may be reluctant to start pharmacological treatment for low-risk stage 1 hypertension, even if lifestyle changes prove insufficient. Patient-centered discussions regarding the benefits of starting antihypertensive medications in this setting will be important to ensure appropriate uptake.

## 6 Conclusion

Application of the 2025 AHA/ACC guidelines for the management of high blood pressure may increase eligibility for hypertension treatment by up to 19.0 million additional adults, depending on the success of lifestyle modification for lower-risk stage 1 hypertension. Increases in treatment eligibility are most pronounced among middle-aged and obese adults. Given the lack of blood pressure goal attainment in those already on treatment, achieving hypertension control in this expanded population will require substantial public health efforts.

## Author agreement

The authors have agreed to the submission of this manuscript – *Implications of the 2025 AHA/ACC High Blood Pressure Guidelines on the Initiation and Intensification of Blood Pressure-Lowering Medications among US Adults* – September 2024 and the materials in this manuscript have not been previously published nor are in consideration for publication elsewhere.

## Disclosures

EDP and AMN receive research support to their institution from Amgen and Esperion. AMN receives consulting fees from Amgen, Arrowhead, Bayer, Esperion, Janssen, Eli Lilly, Merck, New Amsterdam, Novartis, Novo Nordisk, Pfizer, Roche, and Silence Therapeutics and EDP receives consulting fees from Janssen and Novo Nordisk. AS has no disclosures to declare.

## Data sharing statement

The data used for this analysis are publicly available (https://wwwn.cdc.gov/nchs/nhanes/). The related statistical code has also been made publicly available (https://github.com/ahmedsayedcardio/2025_BP_Guidelines_Implications) and the software required for its execution is also freely available (https://www.r-project.org/).

## Sources of funding

This work did not receive external funding.

## CRediT authorship contribution statement

**Ahmed Sayed:** Conceptualization, Formal analysis, Visualization, Writing – original draft. **Eric D. Peterson:** Supervision, Writing – review & editing. **Ann Marie Navar:** Supervision, Writing – review & editing.

## Declaration of competing interest

The authors declare the following financial interests/personal relationships which may be considered as potential competing interests: Ann Marie Navar reports a relationship with Amgen, Arrowhead, Bayer, Esperion, Janssen, Eli Lilly, Merck, New Amsterdam, Novartis, Novo Nordisk, Pfizer, Roche, and Silence Therapeutics that includes: consulting or advisory. Eric D. Peterson reports a relationship with Janssen and Novo Nordisk that includes: consulting or advisory. If there are other authors, they declare that they have no known competing financial interests or personal relationships that could have appeared to influence the work reported in this paper.

## Appendix Supplementary materials

Image, application 1

Image, application 2

## References

[1] Mensah G.A., Fuster V., Murray C.J.L., Roth G.A. (2023). Global Burden of cardiovascular diseases and Risks, 1990-2022. J Am Coll Cardiol.

[2] Jones D.W., Ferdinand K.C., Taler S.J., Johnson H.M., Shimbo D., Abdalla M., Altieri M.M., Bansal N., Bello N.A., Bress A.P. (2025). AHA/ACC/AANP/AAPA/ABC/ACCP/ACPM/AGS/AMA/ASPC/NMA/PCNA/SGIM Guideline for the Prevention, detection, evaluation and management of high blood pressure in adults: a report of the American college of cardiology/American heart association joint committee on clinical practice guidelines. Circulation.

[3] Whelton P.K., Carey R.M., Aronow W.S., Casey D.E., Collins K.J., Dennison Himmelfarb C., DePalma S.M., Gidding S., Jamerson K.A., Jones D.W. (2017). ACC/AHA/AAPA/ABC/ACPM/AGS/APhA/ASH/ASPC/NMA/PCNA Guideline for the prevention, detection, evaluation, and management of high blood pressure in adults: a report of the American college of cardiology/American heart association task force on clinical practice guidelines. Circulation.

[4] Centers for Disease Control and Prevention (CDC). National Center For Health Statistics (NCHS). National Health And Nutrition Examination Survey Data. Hyattsville, MD: U.S. Department Of Health And Human Services, Centers for Disease Control and Prevention, URL: https://wwwn.cdc.gov/nchs/nhanes/.

[5] National Health and Nutrition Examination Survey (2020).

[6] Heeringa S.G., West B.T., Heeringa S.G., Berglund P.A., Berglund P.A. (2017).

[7] R Core Team (2022). https://www.R-project.org/.

[8] Lumley T. (2024).

[9] Valenzuela P.L., Carrera-Bastos P., Galvez B.G., Ruiz-Hurtado G., Ordovas J.M., Ruilope L.M., Lucia A. (2021). Lifestyle interventions for the prevention and treatment of hypertension. Nat Rev Cardiol.

[10] Williams A.R., Wilson-Genderson M., Thomson M.D. (2021). A cross-sectional analysis of associations between lifestyle advice and behavior changes in patients with hypertension or diabetes: NHANES 2015-2018. Prev Med.

[11] Abdalla M., Bolen S.D., Brettler J., Egan B.M., Ferdinand K.C., Ford C.D., Lackland D.T., Wall H.K., Shimbo D., American Heart A. (2023). Implementation strategies to improve blood pressure control in the United States: a scientific statement from the American Heart Association and American Medical Association. Hypertension.

[12] Dhungana R.R., Pedisic Z., de Courten M. (2022). Implementation of non-pharmacological interventions for the treatment of hypertension in primary care: a narrative review of effectiveness, cost-effectiveness, barriers, and facilitators. BMC Prim Care.

[13] Domanski M.J., Wu C.O., Tian X., Hasan A.A., Ma X., Huang Y., Miao R., Reis J.P., Bae S., Husain A. (2023). Association of incident cardiovascular disease with time course and cumulative exposure to multiple risk factors. J Am Coll Cardiol.

[14] Wang N., Harris K., Hamet P., Harrap S., Mancia G., Poulter N., Williams B., Zoungas S., Woodward M., Chalmers J. (2022). Cumulative systolic blood pressure load and cardiovascular risk in patients with diabetes. J Am Coll Cardiol.

[15] Lai C.C., Sun D., Cen R., Wang J., Li S., Fernandez-Alonso C., Chen W., Srinivasan Sathanur R., Berenson Gerald S. (2014). Impact of long-term burden of excessive adiposity and elevated blood pressure from childhood on adulthood left ventricular remodeling patterns. J Am Coll Cardiol.

[16] Ference B.A., Bhatt D.L., Catapano A.L., Packard C.J., Graham I., Kaptoge S., Ference T.B., Guo Q., Laufs U., Ruff C.T. (2019). Association of genetic variants related to combined exposure to lower low-density lipoproteins and lower systolic blood pressure with lifetime risk of cardiovascular disease. JAMA.

[17] Jastreboff A.M., Aronne L.J., Ahmad N.N., Wharton S., Connery L., Alves B., Kiyosue A., Zhang S., Liu B., Bunck M.C. (2022). Tirzepatide once weekly for the treatment of obesity. N Engl J Med.

[18] Kennedy C., Hayes P., Cicero A.F.G., Dobner S., Le Roux C.W., McEvoy J.W., Zgaga L., Hennessy M. (2024). Semaglutide and blood pressure: an individual patient data meta-analysis. Eur Heart J.

[19] Yuan Y., Jin A., Neal B., Feng X., Qiao Q., Wang H., Zhang R., Li J., Duan P., Le Cao (2023). Salt substitution and salt-supply restriction for lowering blood pressure in elderly care facilities: a cluster-randomized trial. Nat Med.

[20] Wang N., Rueter P., Atkins E., Webster R., Huffman M., de Silva A., Chow C., Patel A., Rodgers A. (2023). Efficacy and safety of low-dose triple and quadruple combination pills vs monotherapy, usual care, or placebo for the Initial management of hypertension: a systematic review and meta-analysis. JAMA Cardiol.

[21] Desai A.S., Webb D.J., Taubel J., Casey S., Cheng Y., Robbie G.J., Foster D., Huang S.A., Rhyee S., Sweetser M.T. (2023). Zilebesiran, an RNA interference therapeutic agent for hypertension. N Engl J Med.

[22] Anderson T.S., Wilson L.M., Sussman J.B. (2024). Atherosclerotic cardiovascular disease risk estimates using the predicting risk of cardiovascular disease events equations. JAMA Intern Med.

[23] Zhang Y., An J. (2025). Projected impact of 2025 AHA/ACC high blood pressure guideline on medication use. Hypertension.

