# Supplementary material for: Implications of the 2025 AHA/ACC high blood pressure guidelines on the initiation and intensification of blood pressure-lowering medications among US adults
Source: Am J Prev Cardiol. 2026 Jan 18;25:101400. doi: 10.1016/j.ajpc.2025.101400 (PMC12946893; doi:10.1016/j.ajpc.2025.101400)
Supplement: Supplementary file 2 [file mmc2.docx]

**SUPPLEMENTARY MATERIAL**

**Implications of the 2025 AHA/ACC High Blood Pressure Guidelines on the Initiation and Intensification of Blood Pressure-Lowering Medications among US Adults**

Ahmed Sayed, MD^1^; Eric D. Peterson, MD, MPH^2^; Ann Marie Navar, MD, PhD^2^

^1^ Rochester General Hospital, Rochester, New York

^2^ Department of Medicine, Division of Cardiology, University of Texas Southwestern Medical Center, Dallas, Texas

Supplementary Table 1. Criteria used to define eligibility for pharmacological blood pressure-lowering treatment initiation in the current analysis.

| Criterion | Eligibility for treatment initiation |
| --- | --- |
| SBP ≥140 mm Hg or DBP ≥90 mm Hg in all adults | Eligible for pharmacological treatment initiation by both guidelines |
| SBP ≥130 mm Hg or DBP ≥80 mm Hg in adults with diabetes |  |
| SBP ≥130 mm Hg or DBP ≥80 mm Hg in adults with chronic kidney disease |  |
| SBP ≥130 mm Hg or DBP ≥80 mm Hg in adults with established cardiovascular disease |  |
| SBP ≥130 mm Hg or DBP ≥80 mm Hg in adults aged 65 years or older | Eligible for pharmacological treatment initiation by the 2017 AHA/ACC guidelines |
| SBP ≥130 mm Hg or DBP ≥80 mm Hg in adults with 10-year ASCVD risk of 10% or greater as estimated by the PCE |  |
| SBP ≥130 mm Hg or DBP ≥80 mm Hg in adults with a 10-year ASCVD risk of 7.5% or greater as estimated by PREVENT | Eligible for pharmacological treatment initiation by the 2025 AHA/ACC guidelines |
| SBP ≥130 mm Hg or DBP ≥80 mm Hg in adults with a PREVENT 10-year ASCVD risk of less than 7.5% if a 3-6 month trial of lifestyle modification does not sufficiently reduce BP to <130/80 mm Hg |  |

AHA: American Heart Association; ACC: American College of Cardiology; SBP: Systolic blood pressure; DBP: Diastolic blood pressure; ASCVD: Atherosclerotic cardiovascular disease; PCE: Pooled Cohort Equation; PREVENT: Predicting Risk of Cardiovascular Disease EVENTs

Supplementary Table 2. US adults receiving pharmacological treatment for hypertension, according to 2025 AHA/ACC treatment goal.

| **Variable** | **Strata** | **Blood pressure category** | **Proportion [95% CI], %** | **Number [95% CI], millions** |
| --- | --- | --- | --- | --- |
|  | **Overall** | SBP ≥130 or DBP ≥80 (Intensification to reach <130/80 is recommended) | 55.0 [51.3 to 58.7] | 17.4 [16.2 to 18.5] |
|  |  | 120-129/<80 (Intensification to reach <120/80 is preferred) | 17.6 [14.7 to 20.9] | 5.6 [4.4 to 6.7] |
|  |  | At preferred target (<120/80) | 27.3 [23.1 to 32.0] | 8.6 [6.9 to 10.4] |
| **Age (years)** | **Below 65** | SBP ≥130 or DBP ≥80 (Intensification to reach <130/80 is recommended) | 51.9 [46.5 to 57.2] | 10.6 [9.6 to 11.7] |
|  |  | 120-129/<80 (Intensification to reach <120/80 is preferred) | 17.0 [13.2 to 21.6] | 3.5 [2.5 to 4.5] |
|  |  | At preferred target (<120/80) | 31.1 [26.0 to 36.7] | 6.4 [5.0 to 7.7] |
|  | **65 or older** | SBP ≥130 or DBP ≥80 (Intensification to reach <130/80 is recommended) | 60.9 [55.0 to 66.5] | 6.7 [5.8 to 7.7] |
|  |  | 120-129/<80 (Intensification to reach <120/80 is preferred) | 18.8 [14.9 to 23.4] | 2.1 [1.5 to 2.6] |
|  |  | At preferred target (<120/80) | 20.3 [15.7 to 25.8] | 2.3 [1.6 to 2.9] |
| **Sex** | **Female** | SBP ≥130 or DBP ≥80 (Intensification to reach <130/80 is recommended) | 57.2 [52.6 to 61.7] | 9.9 [9.0 to 10.7] |
|  |  | 120-129/<80 (Intensification to reach <120/80 is preferred) | 15.6 [11.9 to 20.1] | 2.7 [2.0 to 3.4] |
|  |  | At preferred target (<120/80) | 27.2 [21.9 to 33.3] | 4.7 [3.5 to 5.9] |
|  | **Male** | SBP ≥130 or DBP ≥80 (Intensification to reach <130/80 is recommended) | 52.4 [46.8 to 58.0] | 7.5 [6.7 to 8.3] |
|  |  | 120-129/<80 (Intensification to reach <120/80 is preferred) | 20.1 [16.5 to 24.2] | 2.9 [2.2 to 3.6] |
|  |  | At preferred target (<120/80) | 27.5 [22.8 to 32.6] | 3.9 [3.0 to 4.8] |
| **Race and ethnicity** | **NH Black** | SBP ≥130 or DBP ≥80 (Intensification to reach <130/80 is recommended) | 65.7 [60.0 to 70.9] | 2.8 [2.2 to 3.5] |
|  |  | 120-129/<80 (Intensification to reach <120/80 is preferred) | 14.1 [10.5 to 18.5] | 0.6 [0.4 to 0.8] |
|  |  | At preferred target (<120/80) | 20.3 [16.2 to 25.1] | 0.9 [0.6 to 1.2] |
|  | **NH White** | SBP ≥130 or DBP ≥80 (Intensification to reach <130/80 is recommended) | 52.5 [47.4 to 57.6] | 10.9 [9.6 to 12.3] |
|  |  | 120-129/<80 (Intensification to reach <120/80 is preferred) | 17.4 [13.4 to 22.3] | 3.6 [2.5 to 4.7] |
|  |  | At preferred target (<120/80) | 30.1 [24.6 to 36.2] | 6.3 [4.6 to 7.9] |
|  | **NH Asian** | SBP ≥130 or DBP ≥80 (Intensification to reach <130/80 is recommended) | 63.7 [55.3 to 71.4] | 1.0 [0.7 to 1.4] |
|  |  | 120-129/<80 (Intensification to reach <120/80 is preferred) | 18.2 [12.7 to 25.4] | 0.3 [0.2 to 0.4] |
|  |  | At preferred target (<120/80) | 18.1 [13.8 to 23.4] |  |
|  | **Hispanic** | SBP ≥130 or DBP ≥80 (Intensification to reach <130/80 is recommended) | 56.5 [50.0 to 62.8] | 2.1 [1.6 to 2.7] |
|  |  | 120-129/<80 (Intensification to reach <120/80 is preferred) | 20.4 [16.0 to 25.6] | 0.8 [0.5 to 1.0] |
|  |  | At preferred target (<120/80) | 23.1 [18.5 to 28.4] | 0.9 [0.6 to 1.1] |
| **Comorbidities** | **Obesity** | SBP ≥130 or DBP ≥80 (Intensification to reach <130/80 is recommended) | 53.9 [48.1 to 59.6] | 9.5 [8.4 to 10.6] |
|  |  | 120-129/<80 (Intensification to reach <120/80 is preferred) | 18.6 [14.3 to 23.7] | 3.3 [2.4 to 4.2] |
|  |  | At preferred target (<120/80) | 27.6 [21.7 to 34.4] | 4.8 [3.6 to 6.1] |
|  | **Diabetes** | SBP ≥130 or DBP ≥80 (Intensification to reach <130/80 is recommended) | 50.1 [45.1 to 55.2] | 4.1 [3.5 to 4.8] |
|  |  | 120-129/<80 (Intensification to reach <120/80 is preferred) | 16.0 [12.0 to 21.0] | 1.3 [0.9 to 1.7] |
|  |  | At preferred target (<120/80) | 33.9 [27.7 to 40.6] | 2.8 [2.1 to 3.5] |
| **10-year CVD risk** | **Higher risk** | SBP ≥130 or DBP ≥80 (Intensification to reach <130/80 is recommended) | 55.8 [50.7 to 60.9] | 10.0 [8.8 to 11.3] |
|  |  | 120-129/<80 (Intensification to reach <120/80 is preferred) | 18.4 [14.4 to 23.4] | 3.3 [2.4 to 4.2] |
|  |  | At preferred target (<120/80) | 25.7 [20.9 to 31.2] | 4.6 [3.4 to 5.8] |
|  | **Lower risk** | SBP ≥130 or DBP ≥80 (Intensification to reach <130/80 is recommended) | 48.9 [41.3 to 56.6] | 5.7 [4.9 to 6.6] |
|  |  | 120-129/<80 (Intensification to reach <120/80 is preferred) | 18.2 [13.9 to 23.5] | 2.1 [1.5 to 2.8] |
|  |  | At preferred target (<120/80) | 32.8 [26.3 to 40.2] | 3.9 [2.9 to 4.8] |
| ACC: American College of Cardiology; AHA: American Heart Association; CVD: Cardiovascular disease; CKD: Chronic kidney disease | | | | |

**Supplementary Figure 1. Proportion of US adults eligible for initiating or intensifying pharmacological treatment of hypertension according to the 2017 and 2025 AHA/ACC guidelines.** These estimates utilize the expanded PREVENT equations to additionally account for HbA1c and urine albumin-creatinine ratio. Bars represent estimated proportions and error bars represent 95% confidence intervals. For the 2025 guidelines, two sets of estimates depending on whether adults with a low PREVENT-based 10-year risk (i.e., <7.5%) and stage 1 hypertension are able to sufficiently reduce blood pressure to below 130/80 mm Hg.

**
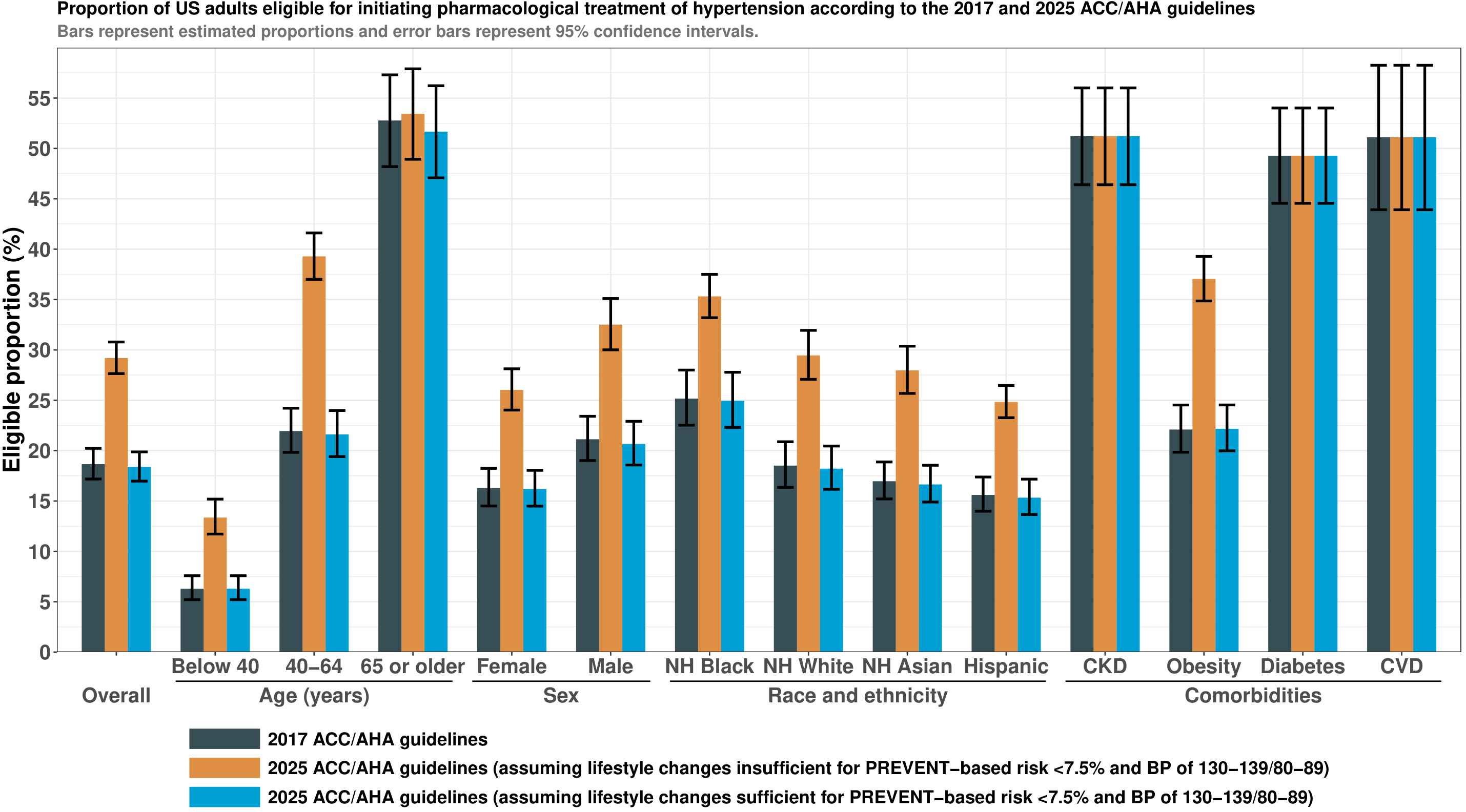
**

**Supplementary Figure 2. Proportion of US adults eligible for initiating or intensifying pharmacological treatment of hypertension according to the 2017 and 2025 AHA/ACC guidelines.** These estimates utilize the expanded PREVENT equations to additionally account for HbA1c (but not urine albumin-creatinine ratio). Bars represent estimated proportions and error bars represent 95% confidence intervals. For the 2025 guidelines, two sets of estimates depending on whether adults with a low PREVENT-based 10-year risk (i.e., <7.5%) and stage 1 hypertension are able to sufficiently reduce blood pressure to below 130/80 mm Hg.

**
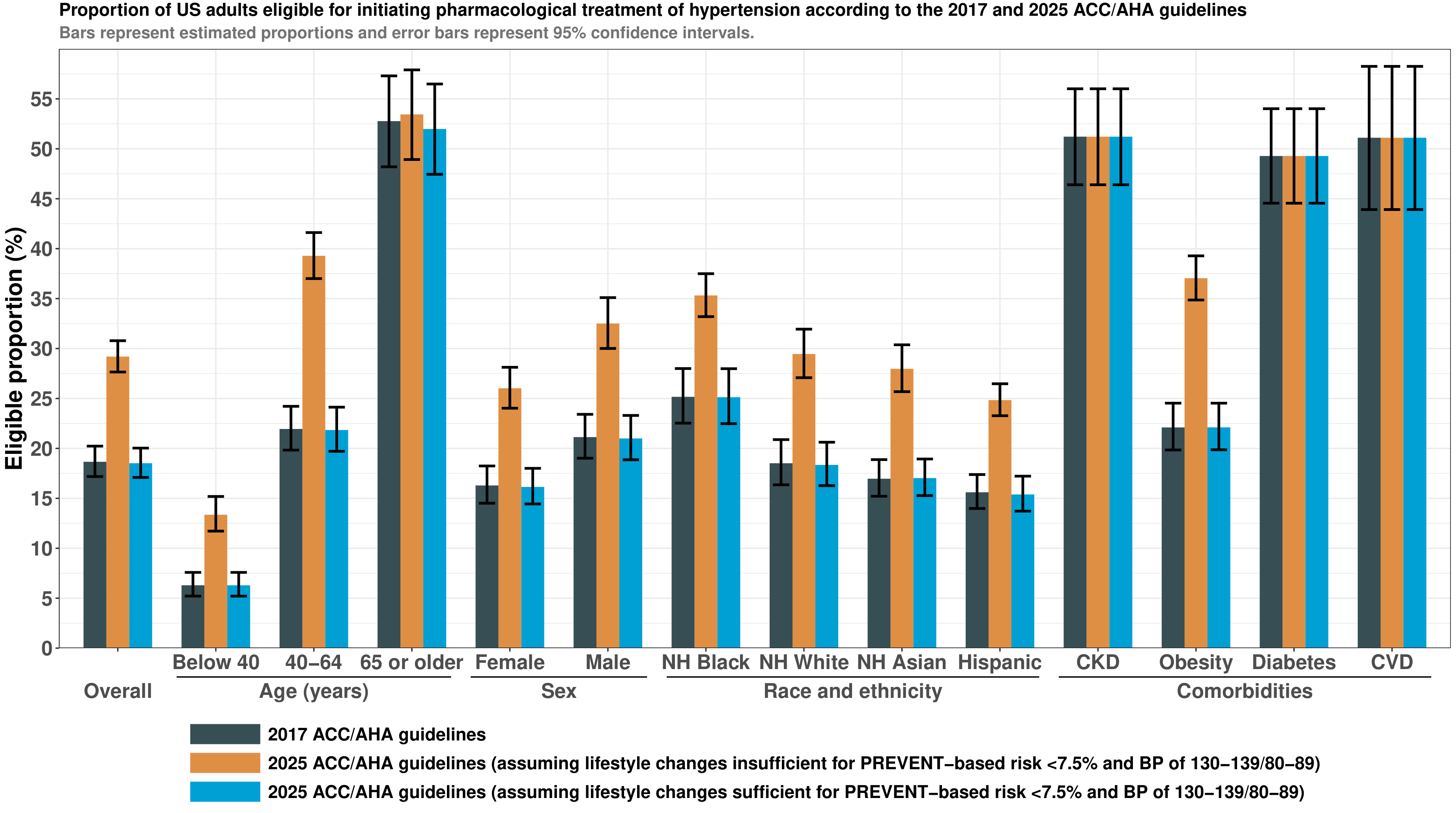
**

**Supplementary Figure 3. Proportion of US adults eligible for initiating or intensifying pharmacological treatment of hypertension according to the 2017 and 2025 AHA/ACC guidelines.** These estimates utilize the expanded PREVENT equations to additionally account for the urine albumin-creatinine ratio (but not HbA1c). Bars represent estimated proportions and error bars represent 95% confidence intervals. For the 2025 guidelines, two sets of estimates depending on whether adults with a low PREVENT-based 10-year risk (i.e., <7.5%) and stage 1 hypertension are able to sufficiently reduce blood pressure to below 130/80 mm Hg.

**
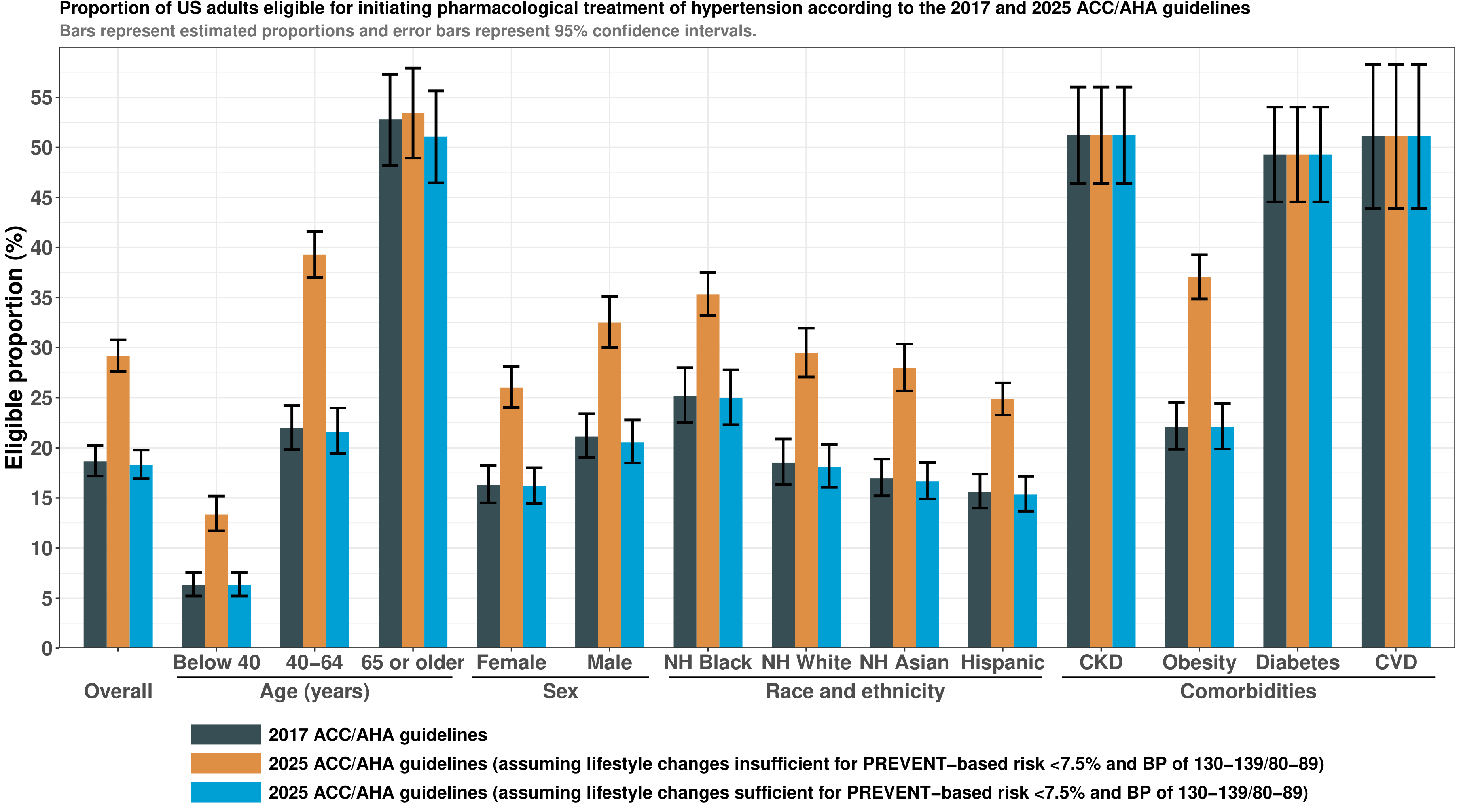
**
