# Supplementary material for: Implications of the 2025 AHA/ACC high blood pressure guidelines on the initiation and intensification of blood pressure-lowering medications among US adults
Source: Am J Prev Cardiol. 2026 Jan 18;25:101400. doi: 10.1016/j.ajpc.2025.101400 (PMC12946893; doi:10.1016/j.ajpc.2025.101400)
Supplement: Supplementary file 1 [file mmc1.docx]

Saturday, December 20, 2025

Erin Michos, MD and Nathan Wong, PhD

Editors, American Journal of Preventive Cardiology

Dear Dr. Michos and Prof. Wong,

We are thankful to have received feedback from the reviewers and editors during our submission to *JACC Advances*. We believe the comments and our responses have enhanced the value of the manuscript of the journal. Below, please find a point-by-point reply to the comments. Reviewer comments are present as numbered points, our replies within this letter are in **bold**, and the additions made to the manuscript that we cite are in red.

Sincerely,

Ahmed Sayed, MBBCh

Ann Marie Navar, MD, PhD

**Reviewer #1**

1. - The authors should be congratulated on their robust attempt to answer a very important question driven by the release of updated hypertension guidelines.

- While a very important question, this seems to have been addressed to some degree by a recent commentary in the journal Hypertension (Hypertension 82(12):p 2064-2066, December 2025). NHANES was similarly used but a marked discrepancy in newly eligible patients (by total number) was found. I would strongly consider reviewing this paper and commenting on how the methodology or results of the current manuscript differ and what additional information is seen in this analysis-of which there are many notable differences worthy of disseminating.

- I am a little curious as to the exact analysis. The 2025 guidelines use PREVENT-CVD as their risk estimation tool with a threshold >7.5% considered elevated risk. In your manuscript (and supplement), your analysis uses PREVENT-ASCVD. Therefore, the analysis itself is flawed by use of a different risk estimation tool than advised by the 2025 guidelines. I would highly advise redoing this entire analysis with PREVENT-CVD versus ASCVD to be concordant with 2025 guidance. Otherwise, there is a significant limitation to your paper that is not being addressed and you would need to address this in the limitation along with explanation as to why you recommended a different risk equation.

**Thank you for citing the aforementioned article (at the time of writing the original manuscript, the article had not yet been released). We have revised the analysis and the major source of discrepancy re: the present estimates and those in the cited paper was the choice of PREVENT-CVD vs PREVENT-ASCVD to determine BP medication eligibility, as the reviewer correctly points out. Our initial motivation in using PREVENT-ASCVD was that prior treatment recommendations based on the PCE were ASCVD-based, and therefore comparing like with like was more appropriate. However, given that treatment recommendations in the 2025 guidelines are based on total CVD risk, and this is ultimately what will dictate recommendations going forward, we have now revised all the analyses herein to use total CVD instead.**

**In addition, we have cited the aforementioned commentary as well and explicitly identified the added value in the present study (Lines 256 to 264):**

**“***A recent commentary by Zhang and An also sought to analyze the projected impact of the 2025 AHA/ACC guideline, finding 26.8 million additional adults may be eligible to initiate pharmacological measures to lower BP if a trial of lifestyle interventions proves insufficient.^1^ Our analysis provides important additional context by identifying that most of this population consists of middle-aged adults, particularly those with obesity. The present analysis also provides data stratified by sex, race/ethnicity, and comorbidity status. Further, we also tackle the burden of above-goal BP among those already taking anti-hypertensive medications, which was beyond the scope of the aforementioned analysis.* **”**

**2.** One aspect of your data is presented in tabular form but should be highlighted. Namely, one of the concerns around the novel PREVENT equation is that it is race agnostic and there have been concerns on how this may impact eligibility for anti-hypertensive therapy. I would strongly consider addressing any change in eligibility by gender and/or race/ethnicity.

**We concur that this is an important point in view of the removal of race coefficients from PREVENT. We have commented explicitly on this as follows (Lines 246 to 255):**

“*An important novel aspect of the 2025 guidelines is the use of the race-agnostic PREVENT. Previous data has shown that PREVENT yielded lower ASCVD risk estimates than PCE, with the difference being largest among Black persons.^2^ These findings lead to concerns that usage of PREVENT would lead to lesser utilization of preventive therapies in Black patients. However, in the present analysis, the percentage change in eligibility was largely similar between Black and White persons (0% and -0.3% respectively). It is important to note that PREVENT-based risk estimates are only relevant for eligibility among adults with stage 1 hypertension, among whom immediate treatment eligibility is determined by 10-year risk estimates. Increases in eligibility by sex were also comparable (-0.1% and -0.4% for females and males respectively).*”

**3.** In your analysis, you use the base equation for PREVENT (albeit ASCVD as above). You then do a follow up analysis using both hemoglobin A1c and UACr. Notably, the intent of multiple PREVENT equations with variable input needs was to make it somewhat modular for those who may not have all of the requisite data points at any given time. Therefore, while I applaud looking at changes between PREVENT base and PREVENT hgbA1c/UACr, I would consider a follow up analysis that looks at just the PREVENT equation with hgbA1c and the PREVENT equation with just UACr (especially as checking UACr in all hypertensive patients is now recommended) and evaluating how this may or may not shift eligibility. Further, when doing this, as above, I would recommend use of PREVENT-CVD to be guideline concordant.

**Thank you for raising this point. We have now added 2 additional analyses wherein we look at numbers with HbA1c and UACR alone as Supplementary Figures 2-3. They are copied below for your convenience:**

**HbA1c:**

**UACR:**

**The corresponding part of the Results reads as follows (Lines 150 to 153):**

*“Sensitivity analyses using the expanded version of PREVENT (to include HbA1c, the urine albumin-creatinine ratio, and both) yielded similar results and are shown in* ***Supplementary Figure 1-3****.”*

**4.** I am curious as to why you used age 20 as the lower bounds of this analysis? The guidelines would apply to age 18 and older and NHANES would cover this age range as well.

**The primary reason was that data on CVD is only available in respondents aged 20 years and older. Because this is an essential part of treatment recommendations (since patients with CVD should be treated to a target <130/80 regardless of PREVENT-based risk estimates), we excluded patients aged 18 or 19 years from the present analysis.**

**5.** For your analysis of intensification, I would consider looking at factors the predispose to who is at goal or not at goal (such as age, gender, race, etc). In terms of your analysis of intensification, could you specify in which groups you considered for this analysis? In the manuscript, it appears that all groups are deemed eligible for analysis in terms of intensification. Yet the guidelines do not state this. The first statement regarding intensification is in those in whom their indication for treatment is based on risk (10 year CVD risk > or <7.5%) with, as you noted, a class 1 indication if higher risk and 2B if lower risk so I do appreciate the subgroup analysis. Similarly, there is a recommendation for intensification with in those with diabetes. For CKD, stroke (after the acute period), PAD, and CAD there is no recommendation for intensification and therefore these patients should be excluded from this analysis.

**Thank you for raising this important point. We have now excluded patients with CKD, stroke, CAD, or HF from the intensification analysis (NHANES does not contain data on PAD). The revised figure is below for your convenience:**

**Data for patients with CKD and CVD has been added separately (Lines 195 to 200):**

***“****Among adults with CVD receiving antihypertensive medications (N = 14.0 million), the proportion with a BP above 130/80 mmHg was 62.5% [95%CI: 58.4 to 66.4%] Among adults with CKD (N = 17.0 million), the proportion with a BP above 130/80 mmHg was 66.6% [95%CI: 61.3 to 71.6%].”*

**We have also explicitly highlighted the groups with the largest percentage of BP above 130/80mmHg as follows (Lines 189 to 191):**

***“****Older persons (60.9%), Non-Hispanic Black persons (65.7%), and Asian persons (63.7%) had the highest prevalence of BP above 130/80 mmHg* ***(Figure 3 and Supplementary Table 2).****”*

**Reviewer #2:**

1. Thank you for the opportunity to review this manuscript evaluating hypertension treatment eligibility under the 2025 AHA/ACC guideline. This manuscript addresses an important question but faces significant issues related to methodological validity, clarity, and interpretive alignment with the 2025 guideline. Most notably, using PREVENT-ASCVD instead of PREVENT-CVD substantially alters risk classification and guidance-based eligibility estimates. Strengthening the introduction, clarifying methods, refining definitions (especially for treatment intensification), contextualizing results within the existing literature, and expanding the discussion of clinical, implementation, and policy implications will greatly improve the manuscript. Below, I provide detailed comments organized by manuscript section to guide revision.

**Thank you for your comments; we have now revised the analysis to use PREVENT-CVD instead of PREVENT-ASCVD. All estimates, figures, and tables have been revised accordingly.**

2. A foundational concern is the use of the PREVENT-ASCVD equation to assign treatment eligibility, whereas the 2025 AHA/ACC Hypertension Guideline uses PREVENT-CVD 10-year total CVD risk to determine Stage-1 treatment initiation. Because PREVENT-ASCVD generates meaningfully lower risk estimates and excludes heart failure outcomes, all treatment-eligibility classifications in the manuscript do not align with the guideline. This misapplication affects every major result including the size and characteristics of the newly eligible population, concordance, and treatment-intensification projections and requires full reanalysis.

   **Thank you for this comment. As outlined in the first response to Reviewer #1, we have now redone the entire analysis using PREVENT-CVD risk estimates instead.**

3. Introduction

Underdeveloped framing of the rationale and evidence base

The introduction provides only brief background on recent guideline changes and does not sufficiently explain:

• the motivation for incorporating PREVENT into the 2025 guideline,
• the distinction between PREVENT-ASCVD and PREVENT-CVD,
• the known limitations of the 2017 framework, or
• how total CVD risk differs clinically and epidemiologically from ASCVD-only risk.

Missing positioning relative to the emerging literature

Recent analyses (including several published and in press) have evaluated PREVENT's impact on population risk distribution and antihypertensive eligibility. The manuscript does not situate its research question in this emerging evidence base, nor does it highlight discrepancies between this study's projected eligibility changes and previously reported findings. This limits contextualization and justification for the study.

**Thank you for these comments. We have now revised the introduction to ensure that it adequately discusses the rationale for PREVENT’s incorporation and key updates compared to the 2017 guidelines (Lines 25 to 42):**

*“Elevated blood pressure remains a leading cause of morbidity and mortality in the US and worldwide.^3^ The newly-introduced 2025 American College of Cardiology/American Heart Association (AHA/ACC) guidelines on the management of high blood pressure in adults contain a number of potentially important changes compared to its 2017 predecessor.^4,5^ The most prominent modifications include the use of the Predicting Risk of Cardiovascular Disease Events (PREVENT) equations rather than the Pooled Cohort Equations (PCE) for risk assessment, the use of total cardiovascular disease (CVD) rather than atherosclerotic cardiovascular disease (ASCVD)-based risk estimates, a new recommendation for pharmacological treatment of lower-risk adults with stage 1 hypertension if lifestyle changes prove insufficient, and a preference for a blood pressure (BP) target of <120/80 rather than <130/80 where clinically feasible.

These changes may materially expand the number of US adults eligible for initiation of pharmacological therapy and also the number of currently-treated US adults eligible for treatment intensification to achieve the new preferred BP targets. Accordingly, we sought to describe the implications of the new recommendations on the management of hypertension among US adults and identify the demographic subgroups where these recommendations are most impactful.”*

4. Use of PREVENT-ASCVD for treatment eligibility As noted above, this is a critical issue. The choice of risk equation must align with guideline-specified outcomes; otherwise, treatment-eligibility estimates are not interpretable.
   **As outlined above, we have now revised the analysis to use PREVENT-CVD risk estimates to ensure concordance with the current recommendations.**

5. Age Range Misaligned With PREVENT Validation and Guideline Application

   The analytic population includes adults aged greater than or equal to 20 years, but PREVENT is validated for ages 30-79 years, and the guideline restricts risk-based Stage-1 treatment decisions to this age range. The manuscript does not clarify how treatment eligibility was determined for younger participants or how non-validated PREVENT scores were handled.

**We understand the reviewer’s concern regarding age eligibility. We only applied PREVENT’s risk estimates to adults aged 30 to 79 years old (and PCE to adults aged 40 to 79 years old) as we did not want to extrapolate their use beyond the demographics in which they were developed. For individuals outside this age range, guideline recommendations are still applicable. The treatment of a 25-year old with a BP of 143/85 (for instance) is not dependent on their risk score, as stage 2 hypertension warrants treatment regardless of estimated 10-year risk. Thus, for adults within the eligible age range for PREVENT/PCE, their risk estimates were used as an additional indication to start pharmacological therapy. For adults outside their eligible age ranges, indications to start pharmacological treatment were not based on risk estimates. We have now clarified this as follows (Lines 82 to 86):**

*“Risk estimates by PREVENT and PCE were only calculated for adults aged 30 to 79 years and 40 to 79 years respectively, as these were the age-groups in which these equations were intended for use. For persons outside these age ranges, treatment recommendations were based on BP only.”*

6. Ambiguity around treatment "intensification" definition

   The manuscript frequently refers to "treatment intensification," but the operational definition is unclear:

   • Does intensification refer to initiating pharmacologic therapy?
   • Does it refer to adjustment of existing therapy?
   • Is intensification being applied to all adults with BP {greater than or equal to}120/80 mm Hg, regardless of risk category or medication status?

   **In the context of the 2025 guidelines and the present manuscript, intensification simply refers to the pursuit of the BP target, regardless of whether it is achieved by increasing dose or adding additional agents. In accordance with the 5^th^ comment from Reviewer #1, this analysis is now restricted to patients with an explicit guideline-based recommendation for the pursuit of a target BP <120/80 mmHg, excluding those with established CVD or CKD.**

7. Several aspects of the methods section require expansion:

   • Handling of missing PREVENT inputs

   **Thank you for raising this point. To ensure that our results could be extrapolated to the US population while mitigating any bias due to missing data, we used inverse-probability weighting (matching on age, sex, and race) to adjust survey weights for adults with non-missing data. This has now been clarified as follows in the Methods (Lines 119 to 128):**

*“**To adjust for item nonresponse (that some survey respondents did not answer questions or have measurements necessary to determine their eligibility), the survey weights of adults with non-missing data were adjusted using their response propensities.^6^ Response propensities for each participant were estimated using a survey-weighted logistic regression model containing age, sex, and race as predictor variables. Survey weights were then adjusted by the inverse of the predicted response probabilities. This allowed us to make inference regarding the target population (US adults aged 20 years or older) despite data missingness while also serving to mitigate biases that may arise due selective non-response.”*

8. Application of survey weights across pooled NHANES cycles
   **We adjusted NHANES weights for each survey cycle using published recommendations, treating the 2017-2020 cycle as being worth 3.2 years of data (compared to the standard 2-year weight used in prior cycles).^7^ This has now been mentioned explicitly as follows:**

*“**Combining survey weights of the 2017-2020 and 2015-2016 survey cycles was done in accordance with published NHANES analytic guidelines.****^7^****”*

9. Whether adults on antihypertensive medication were classified by measured BP or by diagnosis
   **Adults already on antihypertensive medications were treated as having hypertension (by virtue of reporting the use of antihypertensive medications for the explicit purpose of lowering their BP).**

10. How conditional Stage-1 recommendations ("if lifestyle modification is insufficient") were treated analytically vs. in scenario analyses
    **These two scenarios were explicitly analyzed to transparently portray the impact under either scenario. Table 1 has 2 separate headers for recommendations that would be immediately applicable based on current BP levels and recommendations that would be applicable only if lifestyle interventions are insufficient.**

11. How adults with comorbidities (diabetes, CKD, etc.) were handled as they would be eligible for treatment irrespective of their PREVENT score risk
    **Adults with these conditions were considered eligible for BP-lowering medications if their BP was >130/80, regardless of their PREVENT score.**

12. Findings diverge markedly from published PREVENT-aligned analyses

    The manuscript reports a large expansion in eligibility (up to 16.5 million), while multiple other studies including recent NHANES-based analyses (Khan, 2025) have shown only modest shifts when PREVENT-CVD is applied. These discrepancies likely stem from the use of PREVENT-ASCVD, inclusion of younger participants, and the treatment of conditional recommendations as immediate pharmacologic eligibility. The manuscript should acknowledge, reconcile, and contextualize these differences.

    **We understand the reviewer’s concern. However, we can not find an analysis published by Khan et al. specifically investigating the impact of the new BP guidelines on medication eligibility. The one commentary that may be relevant here, that published by Zhang et al (outlined in response to query #1 from the first reviewer),***^1^* **found a sizeable number of potentially eligible additional adults (26.8 million).**

13. Underdeveloped interpretation of findings

    The discussion does not fully engage with:

    • why the observed magnitude of eligibility change differs sharply from prior studies,
    **As outlined in the response above, the current manuscript is in agreement with a prior commentary suggesting a sizeable increase in the number of potentially eligible adults.**

• how use of PREVENT-ASCVD alters risk classification,
**All analyses have been revised to include PREVENT-CVD rather than PREVENT-ASCVD**

• the clinical relevance of the newly eligible phenotype identified, or the implications of applying risk-based treatment across younger or metabolically low-risk individuals.
**This has now been explicitly discussed on Lines 220 to 240 of the Discussion:**

*“A key finding of this study is that middle-aged adults (age 35-55) experienced the greatest expected increase eligibility for pharmacological treatment. Under the prior guideline, these adults did not have sufficiently high risk predicted by the PCE to meet criteria to initiate pharmacological treatment. In contrast, the proportion of older adults eligible to initiate pharmacological treatment is nearly identical under both guidelines, likely because an age >65 was considered a risk-enhancing risk factor under older guidelines that merits treatment initiation in stage 1 hypertension regardless of calculated risk. Multiple studies support the long-term, cumulative risk of prolonged exposure to high blood pressure.^8-11^ Promoting earlier BP-lowering may reduce this cumulative exposure and thus mitigate the long-term cardiovascular risk posed by elevated BP.*

*The reason for the increase in eligibility among people with obesity is twofold. First, persons with obesity are more likely to have stage 1 hypertension, which may merit treatment in the new guidelines even with a lower 10-year predicted risk. Second, unlike the PCE, the PREVENT calculator accounts for BMI and produces higher 10-year risk estimates for persons with obesity. An important related development has been the approval and wider use of effective weight-loss medications which also substantially reduce BP in persons with obesity.^12,13^ These medications, notwithstanding concerns about wider access and long-term affordability, will complement current use of anti-hypertensive medications and help control elevated BP in a substantial proportion of obese people.”*

14. Lack of connection to policy, equity, and implementation
    The guideline's adoption of PREVENT represents a shift toward more equitable and individualized risk assessment. The discussion does not explore:

    • how PREVENT-CVD improves calibration for women, Black adults, or socially disadvantaged populations,
    **In the present study, we did not find large differences between Black and White adults or men and women with respect to changes in medication eligibility. We have now explicitly mentioned this as follows (Lines 248 to 257):**

“*An important novel aspect of the 2025 guidelines is the use of the race-agnostic PREVENT. Previous data has shown that PREVENT yielded lower ASCVD risk estimates than PCE, with the difference being largest among Black persons.^2^ These findings lead to concerns that usage of PREVENT would lead to lesser utilization of preventive therapies in Black patients. However, in the present analysis, the percentage change in eligibility was largely similar between Black and White persons (0% and -0.3% respectively). It is important to note that PREVENT-based risk estimates are only relevant for eligibility among adults with stage 1 hypertension, among whom immediate treatment eligibility is determined by 10-year risk estimates. Increases in eligibility by sex were also comparable (-0.1% and -0.4% for females and males respectively).*”

• the implementation challenges for integrating risk calculators into EHR systems,
**This has now been explicitly mentioned as follows (Lines 276 to 280):**

*“Further, implementation of the new guideline recommendations in daily practice is likely to be subject to several limitations, including clinical inertia challenges and delays in embedding risk calculators within the electronic medical record. As such, the projections studied here are unlikely to be realized to their full extent.”*

• the role of shared decision-making for Stage-1 hypertension
**We have now explicitly tackled this item as follows (Lines 280 to 284):**

*“Furthermore, many physicians and patients may be reluctant to start pharmacological treatment for low-risk stage 1 hypertension, even if lifestyle changes prove insufficient. Patient-centered discussions regarding the benefits of starting antihypertensive medications in this setting will be important to ensure appropriate uptake.”*

• broader public-health or policy implications.
**The public health implications are discussed on Lines 220 to 230:**

*“A key finding of this study is that middle-aged adults (age 35-55) experienced the greatest expected increase eligibility for pharmacological treatment. Under the prior guideline, these adults did not have sufficiently high risk predicted by the PCE to meet criteria to initiate pharmacological treatment. In contrast, the proportion of older adults eligible to initiate pharmacological treatment is nearly identical under both guidelines, likely because an age >65 was considered a risk-enhancing risk factor under older guidelines that merits treatment initiation in stage 1 hypertension regardless of calculated risk. Multiple studies support the long-term, cumulative risk of prolonged exposure to high blood pressure.^8-11^ Promoting earlier BP-lowering may reduce this cumulative exposure and thus mitigate the long-term cardiovascular risk posed by elevated BP.*

*The reason for the increase in eligibility among people with obesity is twofold. First, persons with obesity are more likely to have stage 1 hypertension, which may merit treatment in the new guidelines even with a lower 10-year predicted risk. Second, unlike the PCE, the PREVENT calculator accounts for BMI and produces higher 10-year risk estimates for persons with obesity. An important recent development has been the approval and wider use of effective weight-loss medications which also substantially reduce BP in persons with obesity.^12,13^ These medications, notwithstanding concerns about wider access and long-term affordability, will complement current use of anti-hypertensive medications and help control elevated BP in a substantial proportion of obese people.*

*Our study also highlights the ongoing gaps in hypertension control amongst patients already on therapy. Only around half of patients with hypertension had achieved a BP <130/<80, and barely 25% had achieved the new preferred goal of <120/80. Closing this gap will likely require a multifaceted approach, including public health measures such as wider use of salt-substitutes^14^, optimizing use of currently-available medications such as by wider use of fixed-dose combination pills,^15^ and the use of novel long-acting treatment options where clinically appropriate.^16^ ”*

15. Key limitations including use of a non-guideline risk model, age-range mismatch, and ambiguity around intensification methodology are not sufficiently acknowledged.
    **As outlined above, we have now revised all risk estimates to use PREVENT-CVD, clarified how adults outside the eligible age range were handled, and clarified the term intensification.**

**References:**

1.	Zhang Y, An J. Projected Impact of 2025 AHA/ACC High Blood Pressure Guideline on Medication Use. *Hypertension*. Dec 2025;82(12):2064-2066. doi:10.1161/HYPERTENSIONAHA.125.25903

2.	Anderson TS, Wilson LM, Sussman JB. Atherosclerotic Cardiovascular Disease Risk Estimates Using the Predicting Risk of Cardiovascular Disease Events Equations. *JAMA Intern Med*. Aug 1 2024;184(8):963-970. doi:10.1001/jamainternmed.2024.1302

3.	Mensah GA, Fuster V, Murray CJL, Roth GA. Global Burden of Cardiovascular Diseases and Risks, 1990-2022. *J Am Coll Cardiol*. Dec 19 2023;82(25):2350-2473. doi:10.1016/j.jacc.2023.11.007

4.	Jones DW, Ferdinand KC, Taler SJ, et al. 2025 AHA/ACC/AANP/AAPA/ABC/ACCP/ACPM/AGS/AMA/ASPC/NMA/PCNA/SGIM Guideline for the Prevention, Detection, Evaluation and Management of High Blood Pressure in Adults: A Report of the American College of Cardiology/American Heart Association Joint Committee on Clinical Practice Guidelines. *Circulation*. Aug 14 2025;doi:10.1161/CIR.0000000000001356

5.	Whelton PK, Carey RM, Aronow WS, et al. 2017 ACC/AHA/AAPA/ABC/ACPM/AGS/APhA/ASH/ASPC/NMA/PCNA Guideline for the Prevention, Detection, Evaluation, and Management of High Blood Pressure in Adults: A Report of the American College of Cardiology/American Heart Association Task Force on Clinical Practice Guidelines. *Circulation*. Oct 23 2018;138(17):e484-e594. doi:10.1161/CIR.0000000000000596

6.	Heeringa, S.G., West, B.T., Heeringa, S.G., Berglund, P.A., & Berglund, P.A. (2017). Applied Survey Data Analysis (2nd ed.). Chapman and Hall/CRC. <https://doi.org/10.1201/9781315153278>.

7.	National Health and Nutrition Examination Survey, 2017–March 2020 Prepandemic File: Sample Design, Estimation, and Analytic Guidelines Data Evaluation and Methods Research.

8.	Domanski MJ, Wu CO, Tian X, et al. Association of Incident Cardiovascular Disease With Time Course and Cumulative Exposure to Multiple Risk Factors. *J Am Coll Cardiol*. Mar 28 2023;81(12):1151-1161. doi:10.1016/j.jacc.2023.01.024

9.	Wang N, Harris K, Hamet P, et al. Cumulative Systolic Blood Pressure Load and Cardiovascular Risk in Patients With Diabetes. *J Am Coll Cardiol*. Sep 20 2022;80(12):1147-1155. doi:10.1016/j.jacc.2022.06.039

10.	Lai C-C, Sun D, Cen R, et al. Impact of Long-Term Burden of Excessive Adiposity and Elevated Blood Pressure From Childhood on Adulthood Left Ventricular Remodeling Patterns. *J Am Coll Cardiol*. 2014/10/14 2014;64(15):1580-1587. doi:10.1016/j.jacc.2014.05.072

11.	Ference BA, Bhatt DL, Catapano AL, et al. Association of Genetic Variants Related to Combined Exposure to Lower Low-Density Lipoproteins and Lower Systolic Blood Pressure With Lifetime Risk of Cardiovascular Disease. *JAMA*. Oct 8 2019;322(14):1381-1391. doi:10.1001/jama.2019.14120

12.	Jastreboff AM, Aronne LJ, Ahmad NN, et al. Tirzepatide Once Weekly for the Treatment of Obesity. *N Engl J Med*. Jul 21 2022;387(3):205-216. doi:10.1056/NEJMoa2206038

13.	Kennedy C, Hayes P, Cicero AFG, et al. Semaglutide and blood pressure: an individual patient data meta-analysis. *Eur Heart J*. Oct 7 2024;45(38):4124-4134. doi:10.1093/eurheartj/ehae564

14.	Yuan Y, Jin A, Neal B, et al. Salt substitution and salt-supply restriction for lowering blood pressure in elderly care facilities: a cluster-randomized trial. *Nature Medicine*. 2023/04/01 2023;29(4):973-981. doi:10.1038/s41591-023-02286-8

15.	Wang N, Rueter P, Atkins E, et al. Efficacy and Safety of Low-Dose Triple and Quadruple Combination Pills vs Monotherapy, Usual Care, or Placebo for the Initial Management of Hypertension: A Systematic Review and Meta-analysis. *JAMA Cardiology*. 2023;8(6):606-611. doi:10.1001/jamacardio.2023.0720

16.	Desai AS, Webb DJ, Taubel J, et al. Zilebesiran, an RNA Interference Therapeutic Agent for Hypertension. *N Engl J Med*. Jul 20 2023;389(3):228-238. doi:10.1056/NEJMoa2208391
